# LEM-Domain-Containing Inner Nuclear Membrane Proteins: Emerging Regulators of Intranuclear Signaling

**DOI:** 10.3390/ijms27020942

**Published:** 2026-01-17

**Authors:** Byongsun Lee, Hyunggeun Lee, Jaekyung Shim

**Affiliations:** Department of Bioresources Engineering, Sejong University, Seoul 05006, Republic of Korea; coolbs@sju.ac.kr (B.L.); prosperoot0508@sju.ac.kr (H.L.)

**Keywords:** LEM-domain proteins, inner nuclear membrane, intranuclear signaling, nuclear lamina, transcription factor regulation, signal transduction, LAP2, emerin, MAN1, muscular dystrophy

## Abstract

The LAP2–emerin–MAN1-domain (LEM-D) proteins constitute a family of inner nuclear membrane proteins that play essential roles in the spatial regulation of intranuclear signaling. Defined by the conserved LEM domain, these proteins interact with chromatin, nuclear lamins, and barrier-to-autointegration factor (BAF), thereby linking nuclear architecture to signal-dependent transcriptional control. This review summarizes current knowledge on the structural features and molecular functions of representative LEM-D proteins, including LAP2, emerin, and MAN1, with a particular focus on their emerging roles as regulators of intranuclear signaling pathways. We discuss how these proteins modulate the activity of transcription factors involved in Hedgehog, Wnt/β-catenin, STAT3, Notch, and transforming growth factor-β (TGF-β) signaling by temporally retaining them at the inner nuclear membrane and controlling their access to chromatin. Furthermore, this review highlights the physiological and pathological relevance of LEM-D-mediated signaling regulation, especially in the context of muscle development, regeneration, and nuclear envelope-associated diseases such as muscular dystrophies. By integrating structural, signaling, and disease-related perspectives, this review proposes a conceptual framework in which LEM-D proteins function as critical intranuclear signaling hubs. Understanding these mechanisms provides new insights into nuclear signal transduction and suggests potential therapeutic targets for diseases associated with nuclear envelope dysfunction.

## 1. Introduction

The inner nuclear membrane (INM) plays a crucial role in maintaining nuclear envelope integrity and regulating essential cellular functions, including gene expression, nuclear assembly, and structural organization [1,2,3,4,5,6]. Among the proteins associated with the INM, the LAP2–emerin–MAN1 domain (LEM-D)-containing proteins represent a conserved family that includes LAP2, emerin, and MAN1 [1,2]. These proteins contribute not only to the mechanical stability of the nucleus but also to regulatory processes that shape cellular physiology through interactions with chromatin and signaling molecules [2,3].

The INM is distinguished by its unique molecular composition and organization supported by intermediate filament proteins known as lamins that form the nuclear lamina beneath the membrane [2,4,5]. This lamina provides structural support while anchoring peripheral chromatin regions, thereby influencing genome organization and transcriptional regulation [4,6,7]. Within this framework, LEM-D proteins are prominent components that link nuclear architecture to functional regulation at the nuclear periphery.

**Figure 1:**
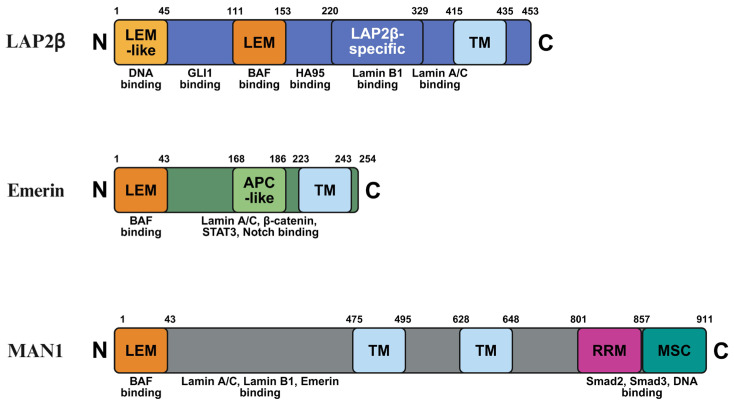
**Schematic representation of the domain architecture of LAP2β, emerin, and MAN1.** Each protein is shown from the N-terminus to the C-terminus with amino acid positions indicated. Conserved and functional domains are highlighted, including the LAP2–emerin–MAN1 (LEM) domain, transmembrane (TM) domain, adenomatous polyposis coli-like (APC-like) domain, RNA recognition motif (RRM), and the MAN1 Smad-binding C-terminal (MSC) domain. Known binding partners associated with each domain are indicated below the schematics, illustrating the structural basis for interactions with chromatin, lamins, and signaling-related transcription factors. Created in BioRender. H.L. (2026) https://BioRender.com/tqotmdy.

LEM-D proteins are characterized by a conserved ~40-residue LEM motif that mediates interaction with BAF, thereby linking chromatin to the nuclear periphery and contributing to nuclear architecture [2,6,8] (Figure 1). Emerin, one of the most extensively studied LEM-D proteins, localizes primarily to the INM and contributes to chromatin tethering to the nuclear lamina [9]. Its interactions with BAF and lamins are critical for maintaining nuclear envelope integrity [2,4,9,10]. Importantly, the localization of LEM-D proteins is dynamic during mitosis, enabling their coordinated contribution to nuclear envelope disassembly and reassembly following cell division [11,12].

Beyond structural roles, LEM-D proteins also exhibit regulatory functions with direct implications for gene expression and signaling pathways. Accumulating evidence supports a model in which LEM-D proteins function as spatial regulators that organize signaling complexes at the nuclear periphery, thereby modulating transcription factor accessibility to chromatin rather than acting as passive scaffolds. Structural studies have provided important molecular insight into how LEM-D proteins engage specific binding partners and support this regulatory paradigm [2].

At the molecular level, the LEM motif itself has been structurally characterized, supporting its role as a conserved interaction module shared among multiple INM proteins [13]. For LAP2α, the structural basis of dimerization has been resolved, providing insight into how LAP2α may assemble and scaffold regulatory complexes within the nucleoplasm [14]. In emerin-associated assemblies, structural analysis of the lamin A/C–BAF–emerin ternary complex have identified specific interfaces, disruption of which has been linked to autosomal recessive progeroid disease [15]. In the case of MAN1, detailed structural and biophysical studies have defined the Smad–MAN1 interaction at the INM. Nuclear magnetic resonance (NMR) analyses indicate that the C-terminal RRM-like region characterized as a U2AF homology motif (UHM), which is adapted for protein–protein interactions through a hydrophobic pocket that recruits SMAD proteins [16,17]. Collectively, these structural data are consistent with the concept that LEM-D proteins act as organized platforms integrating nuclear architecture with signal-dependent transcriptional control.

Although a substantial portion of mechanistic evidence linking LEM-D proteins to transcriptional regulation has been generated in skeletal muscle models, accumulating studies support broader, tissue-dependent roles of INM proteins. In particular, LAP2 isoforms have been implicated in regulatory programs beyond myogenesis, including neural differentiation, and LAP2 dysregulation has been associated with non-muscle pathologies such as cancer [18,19,20,21,22,23,24,25]. Likewise, emerin-linked diseases frequently display prominent cardiac involvement, and cardiomyopathy may precede skeletal muscle symptoms in EDMD patients [26,27,28,29,30,31,32]. Moreover, several signaling mediators discussed here exhibit context-dependent outputs across tissues, exemplified by STAT3, which promotes skeletal muscle atrophy in muscle disease settings but can act as an oncogene in non-muscular cells [33,34,35]. For MAN1, regulatory functions extend to developmental and vascular contexts, including TGFβ-dependent vasculogenesis in the embryonic yolk sac [36] and BMP/Smad modulation in embryos [37], while comparative interactome analyses further suggest a unique role for LEM2 in nucleotide excision repair [38]. Collectively, these observations support the concept that nuclear envelope proteins can exert tissue-specific functions depending on cellular state, mechanical environment, and the available signaling networks [39].

Based on these structural and organizational principles, the following sections summarize how individual LEM-D proteins—LAP2, emerin, and MAN1—regulate distinct intranuclear signaling pathways, with particular emphasis on their roles in muscle physiology and nuclear envelope-associated disease.

## 2. LAP2 Isoforms as Spatial Gatekeepers of Intranuclear Signaling

The regulation of intranuclear signaling is a critical aspect of cellular function and gene expression, and the lamina-associated polypeptide 2 (LAP2) family plays a significant role in this process [18,40,41]. LAP2 proteins are integral components of the nuclear envelope, engaging in diverse interactions that affect transcription factor distribution, gene expression, and cellular differentiation [40,42]. In addition to their regulatory functions, LAP2 proteins are well recognized for their structural roles within the nuclear lamina [40,43]. By facilitating the anchoring of chromatin to the inner nuclear membrane (INM), LAP2 proteins contribute to the spatial organization of the nucleus, which is essential for coordinated transcriptional regulation and signaling [41,43].

Multiple LAP2 isoforms, including LAP2α, LAP2β, and LAP2γ, display distinct subnuclear localizations and functional properties that underlie their diverse roles within the nucleus [43,44]. For instance, LAP2β exhibits properties that reinforce the nuclear structure by binding to chromatin, whereas LAP2α interacts with nucleoplasmic lamins to regulate protein stability and localization [42,45]. In addition, LAP2 participates in chromatin regulation through its interaction with barrier-to-autointegration factor (BAF) and the chromatin-associated regulatory protein HA95 [46,47,48,49]. These structural interactions are critical for creating a nuclear environment that supports efficient intranuclear signaling [8,44].

Within this organized nuclear architecture, transcription factors must navigate a highly structured nuclear landscape to exert their regulatory functions [50]. LAP2 proteins have been shown to influence both the localization and activity of multiple transcription factors, most notably within the Hedgehog signaling pathway [41,51,52]. LAP2β associates with GLI1 and histone deacetylase 1 (HDAC1), thereby coupling the acetylation status of GLI1 to its subnuclear distribution. Acetylated GLI1 is transcriptionally attenuated and preferentially retained at the INM, whereas HDAC1-mediated deacetylation promotes GLI1 release into the nucleoplasm and activation of Hedgehog target genes [42,51,53,54,55,56]. Consistent with tissue-context dependence, Gli1 expression marks a sentinel population of muscle stem cells during regeneration, suggesting that LAP2-dependent GLI1 gating may contribute to myogenic repair programs [57,58]. This acetylation-dependent gating mechanism underscores LAP2’s dual role as both a structural scaffold and an active regulator of transcriptional signaling [44,51].

Consistent with tissue-context dependence, Gli1 expression marks a sentinel population of muscle stem cells during regeneration, suggesting that LAP2-dependent GLI1 spatial regulation may contribute to myogenic repair programs [57,58]. These observations support the concept that LAP2-mediated intranuclear signaling is not static but dynamically integrated with tissue-specific transcriptional demands. Beyond Hedgehog signaling, LAP2 isoforms interact with a wide range of nuclear proteins involved in intranuclear signaling and transcriptional regulation [19,59,60]. Notably, LAP2 interacts with the retinoblastoma (Rb) protein, a central regulator of cell cycle progression and gene expression [59,60]. This interaction is required for proper Rb localization and phosphorylation, thereby directly influencing Rb activity and downstream transcriptional programs [59,60].

In addition to Rb, LAP2 isoforms contribute to the stability of nuclear complexes that regulate chromatin architecture and accessibility, processes that are fundamental to gene regulation and cellular responsiveness to signaling cues [14,61,62]. Accumulating evidence indicates that LAP2 also modulates the activity of transcription factors involved in lineage-specific differentiation pathways, including myogenic and neural differentiation programs [18,19,20,21]. LAP2α, in particular, has been shown to facilitate myogenic gene expression by coordinating the spatial organization of gene loci critical for muscle development [19,41,63]. These findings highlight how LAP2 function as a component of broader regulatory networks governing cell fate decisions.

Dysregulation of LAP2 and its associated signaling pathways has been linked to multiple pathological conditions, including cancer and muscular dystrophies [22,23,24,25,64]. Altered LAP2 expression or mutations affecting LAP2 functions can disrupt the balance of intranuclear signaling, leading to aberrant transcriptional programs associated with malignant transformation [25,64]. Similarly, loss of LAP2 functions has been implicated in specific myopathies, where its critical role in muscle gene regulation becomes particularly evident [60,65,66].

Given its central position at the intersection of nuclear architecture and intranuclear signaling, LAP2 represents a potential therapeutic target in diseases characterized by disrupted nuclear signaling dynamics [41,51,55,63]. A detailed understanding of the molecular interactions and regulatory mechanisms involving LAP2 may inform strategies aimed at restoring proper nuclear organization and transcriptional control. Modulation of LAP2 interactions or expression levels could therefore provide a means to alleviate pathological consequences arising from its dysregulation.

## 3. Emerin-Mediated Spatial Control of STAT3 and Notch Signaling

Emerin is an integral inner nuclear membrane protein that has garnered considerable attention due to its essential roles in maintaining nuclear integrity and regulating intranuclear signaling pathways [67,68,69,70,71,72,73]. Mutations in the *EMD* gene encoding emerin are associated with Emery-Dreifuss muscular dystrophy (EDMD), a degenerative disorder characterized by skeletal muscle wasting and cardiac abnormalities [74]. Given its dual structural and regulatory functions, emerin has emerged as a key component linking nuclear architecture to signal-dependent transcription.

Emerin localizes predominantly to the inner nuclear membrane, where it is integrated into the nuclear lamina network composed of intermediate filament proteins that provide mechanical support to the nucleus [75,76,77]. Through interactions with lamins and chromatin-associated factors, emerin contributes to chromatin tethering at the nuclear periphery and participates in mechanotransduction, enabling cells to sense and respond to mechanical cues [78,79,80,81]. This structural role is particularly critical in muscle cells, which experience substantial mechanical strain during contraction and therefore require robust nuclear envelope integrity to maintain cellular homeostasis [4,82,83].

Beyond its architectural functions, emerin plays a prominent role in regulating intranuclear signaling pathways, most notably the STAT3 signaling cascade [68]. STAT3 is a multifunctional transcription factor involved in inflammation, cell growth, and survival, and its nuclear activity is subject to spatial regulation by emerin [68,84,85,86]. Experimental evidence indicates that emerin attenuates STAT3 signaling by retaining STAT3 at the INM, thereby delaying its access to chromatin and transcriptional activation [68]. This regulatory mechanism has been implicated in skeletal muscle homeostasis and suggests a potential point of intervention in conditions associated with aberrant STAT3 activity.

Importantly, available data supports an association between altered emerin–STAT3 regulation and EDMD-related phenotypes, while a direct causal link to disease onset has not yet been established [68,74]. This distinction underscores the need to interpret emerin-mediated signaling defects within the broader context of nuclear envelope dysfunction and tissue-specific susceptibility.

Emerin exerts its regulatory effects through extensive interactions with other nuclear envelope proteins, particularly lamins and BAF [15,71]. Lamins, which form the structural backbone of the nuclear lamina, play central roles in chromatin organization and transcriptional regulation [7,87,88]. Mutations in the *LMNA* (Lamin A/C) cause Hutchinson-Gilford Progeria Syndrome (HGPS), a disorder marked by defective nuclear architecture, impaired muscle development, and severe cardiovascular complications that often lead to premature death [26,27]. Loss of Lamin A/C has been associated with abnormal emerin localization and signaling dysregulation in EDMD-related contexts [6,67,68].

Consistent with the functional importance of emerin–lamin interactions, clinical studies have demonstrated that cardiomyopathy can precede skeletal muscle symptoms in EDMD patients, indicating that cardiac involvement may represent an early manifestation of disease [28,29,30,31]. This observation has important implications for diagnostic evaluation and reinforces the concept that emerin-dependent signaling regulation operates in a tissue-dependent manner [32].

In addition to lamins, emerin interacts with BAF, a chromatin-associated protein essential for nuclear assembly and stability [6,15]. Mutations in *BANF1* (BAF) result in nuclear envelope disruption, activation of DNA damage responses, and cellular senescence, leading to muscle atrophy and progeroid features characteristic of Néstor–Guillermo Progeria Syndrome (NGPS) [89,90,91]. BAF is required for anchoring emerin to the inner nuclear membrane, thereby enabling emerin to coordinate nuclear structure with transcriptional regulation through chromatin architecture modulation [3,67,68,92].

Recent studies have further highlighted the involvement of emerin in signaling pathways governing muscle regeneration, particularly the Wnt and Notch pathways [67,68,69]. Dysregulation of these pathways in the context of emerin deficiency has been linked to impaired muscle differentiation and regeneration, emphasizing the importance of precise spatial control of intranuclear signaling in muscle tissue [74,93]. Together, these findings position emerin as a central integrator of structural and signaling functions at the nuclear envelope.

In summary, emerin functions as a multifunctional regulator that couples nuclear architecture to intranuclear signaling through interactions with STAT3, lamins, BAF, and developmental signaling pathways. While current evidence supports strong associations between emerin-mediated signaling defects and EDMD-related phenotypes, further studies are required to define causal mechanisms. Continued investigation of emerin’s regulatory roles will advance our understanding of muscle pathophysiology and may inform therapeutic strategies targeting nuclear envelope-associated signaling dysfunction.

## 4. MAN1 as a Nuclear Envelope Modulator of TGF-β/Smad Signaling

The study of intranuclear signaling has emerged as a pivotal area of research, revealing how nuclear envelope-associated proteins coordinate complex cellular processes. Among these proteins, MAN1 stands out as a key regulatory component of the inner nuclear membrane (INM) with multifaceted roles in signal-dependent transcription [5,43,94]. This section focuses on the structural features of MAN1 and its function as a modulator of transforming growth factor-β (TGF-β) signaling through interactions with Smad transcriptional regulators.

MAN1, also known as LEM domain-containing protein 3 (LEMD3), is integral INM protein characterized by the presence of a conserved LEM domain that mediates interactions with other nuclear envelope components [95,96]. In addition to this domain, MAN1 contains a nucleoplasmic C-terminal domain that directly engages with Smad proteins, which are vital mediators of TGF-β signaling [16,17,36,97,98]. The protein architecture further includes a carboxyl-terminal RNA recognition motif (RRM), underscoring the structural complexity of MAN1 and its capacity to interact with multiple nuclear factors [38,99].

Structural studies using nuclear magnetic resonance have provided critical insight into the molecular basis of MAN1–Smad interactions. These analyses revealed that the C-terminal RRM of MAN1 is more accurately classified as a U2AF homology motif (UHM), rather than a classical RNA-binding RRM. This UHM is specifically optimized for protein–protein interactions by forming a hydrophobic pocket that recruits SMAD proteins, thereby antagonizing TGF-β signaling at the nuclear periphery [16,17]. This structural specialization enables MAN1 to function as a spatial regulator rather than a simple transcriptional repressor.

The TGF-β signaling pathway plays fundamental roles in cell proliferation, differentiation, and apoptosis [100,101,102]. MAN1 has been identified as a negative regulator of this pathway through its ability to sequester Smad2 and Smad3 at the inner nuclear membrane, thereby limiting their transcriptional activity in response to TGF-β stimulation [16,36,37,99]. Through interactions with R-Smad proteins, MAN1 functions as a molecular gatekeeper that restrains excessive signaling output and helps maintain cellular homeostasis [16,99]. Notably, MAN1 has been shown to interact with all R-Smads except Smad4, highlighting its specificity in modulating Smad-dependent transcription.

Beyond its canonical role in TGF-β signaling, MAN1 interacts with a broad range of nuclear regulatory proteins, reflecting its versatility in intranuclear signaling networks [40,95,103,104]. MAN1 may sequester R-Smads at the nuclear envelope or physically impede their binding to target gene promoters, thereby fine-tuning transcriptional responses [39]. Consistent with this regulatory role, loss of MAN1 function has been associated with hyperactivation of Smad signaling and skeletal abnormalities, including increased bone density [97]. MAN1 has also been implicated in regulating the expression of cytoskeletal proteins and transcription factors involved in angiogenesis, a process essential for normal development and wound repair [68].

Dysregulation of MAN1-dependent signaling has been linked to pathological conditions, most notably cancer, in which aberrant TGF-β signaling contributes to tumor progression and metastasis [38,105,106,107]. These observations underscore the importance of precise MAN1-mediated control of Smad signaling in maintaining normal cellular behavior. Understanding how MAN1 integrates nuclear envelope architecture with transcriptional regulation provides opportunities for therapeutic intervention. Modulating MAN1 expression or function has been proposed as a strategy to restore balanced TGF-β signaling and potentially suppress disease-associated transcriptional programs [99,106]. Recent studies further suggest that targeting MAN1-mediated pathways may offer novel approaches for treating disorders driven by dysregulated TGF-β signaling.

## 5. LEM-Domain Proteins and Muscle Disease

LEM-domain proteins, including LAP2, emerin, and MAN1, play central roles in maintaining nuclear structure and coordinating gene regulatory programs that are particularly critical in mechanically active tissues such as skeletal muscle [2,9]. Through their integration into the nuclear envelope, these proteins link nuclear architecture to intranuclear signaling pathways that govern muscle development, maintenance, and regeneration.

### 5.1. LAP2 and Muscle-Specific Nuclear Regulation

LAP2 is an essential component of the nuclear envelope that contributes to nuclear organization and structural stability through interactions with A-type lamins [108,109]. This interaction provides a scaffold that supports both nuclear architecture and the spatial organization of chromatin, thereby influencing gene expression programs required for muscle function [108,109]. In muscle cells, LAP2 participates in the mechanical response of the nucleus to contractile forces, helping preserve nuclear integrity under repetitive mechanical stress [108,110].

Beyond its structural role, LAP2 has been identified as a transcriptional co-regulator, that influences the expression of muscle-specific genes involved in myogenesis and muscle differentiation [63,108]. During muscle development and regeneration, precise regulation of transcriptional programs is required to coordinate satellite cell activation, cell cycle progression, and differentiation [63,66,108,111]. By modulating transcription accessibility and chromatin organization, LAP2 contributes to the integration of signaling pathways that drive muscle lineage commitment and regenerative capacity.

### 5.2. Emerin and Nuclear Envelope–Associated Muscular Dystrophies

Emerin, encoded by the EMD gene on the X chromosome, is a highly conserved LEM-domain protein that interacts with multiple components of the nuclear lamina, including BAF and Lamins A/C [71,74,112,113,114]. Mutations affecting these interacting partners are strongly associated with nuclear envelope-related pathologies. For example, pathogenic variants in BAF are known to cause NGPS, while mutations in LMNA underlie HGPS as well as several forms of muscular dystrophy [89,90,91,115,116]. These observations underscore the importance of the emerin–BAF–lamin axis in preserving nuclear structural integrity and muscle tissue homeostasis.

Emerin plays a key role in tethering chromatin to the nuclear envelope, a function essential for maintaining nuclear architecture and responsiveness to cellular signaling cues. Its involvement in transcriptional regulation is particularly relevant in muscle, as emerin interacts with multiple transcription factors that influence muscle-specific and cardiac gene expression [67,69,70,73]. Emerin-deficient muscle fibers exhibit characteristic pathological features, including centralized nuclei, fibrosis, and impaired regenerative capacity [117].

At the molecular level, loss of emerin disrupts signaling pathways that are critical for myogenic differentiation and muscle repair [118]. Emerin-deficient myogenic progenitors display aberrant regulation of Wnt, IGF-1, TGF-β, and Notch signaling, collectively leading to compromised muscle regeneration [67,68,69,74,96]. In X-linked Emery–Dreifuss muscular dystrophy (X-EDMD), emerin deficiency is therefore associated with dysregulation of multiple intranuclear signaling pathways rather than a defect in a single signaling axis.

Transcriptional profiling studies further support this integrative role of emerin in muscle biology. Emerin suppresses the transcription of genes associated with Notch, Wnt, and STAT signaling pathways and promotes nuclear envelope localization of LMO7, a transcriptional activator of muscle differentiation, thereby attenuating its transcriptional activity [70]. These regulatory effects are particularly relevant during myogenesis where the temporal balance between Notch and Wnt signaling orchestrates the progression of muscle precursor cells along the myogenic lineage [67,119].

**Figure 2:**
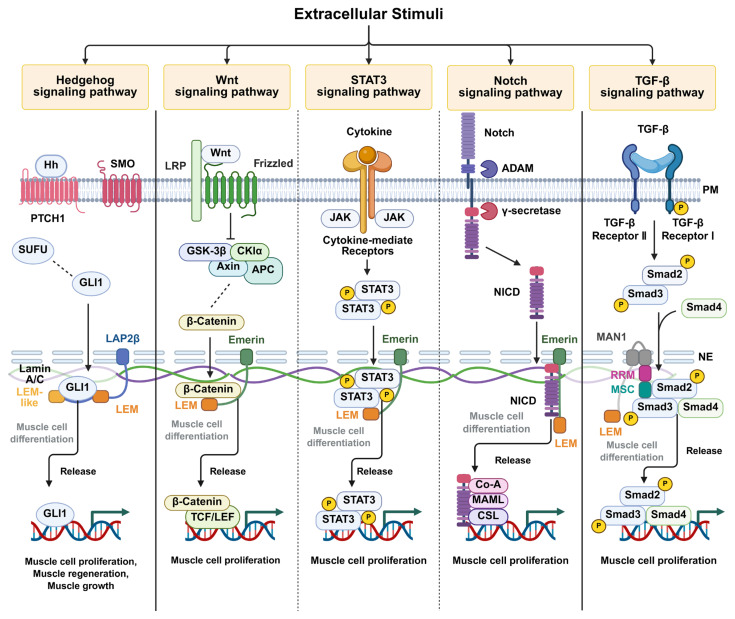
**Proposed model illustrating the regulation of intranuclear signaling pathways by LEM-domain-containing inner nuclear membrane proteins.** LEM-domain proteins, including LAP2β, emerin, and MAN1, interact with transcription factors that translocate into the nucleus through specific domains described in Figure 1. These interactions spatially retain transcription factors at the inner nuclear membrane, thereby modulating their transcriptional activity. Signaling pathways mediated by Hedgehog, Wnt/β-catenin, STAT3, Notch, and transforming growth factor-β (TGF-β) are depicted. Pathways predominantly regulated by emerin are indicated by dotted lines. Transcription factor sequestration at the nuclear envelope is shown in gray, whereas transcriptional activation following release and DNA binding is shown in black. Created in BioRender. H.L. (2026) https://BioRender.com/akzi7ew.

STAT3 signaling also illustrates the context-dependent consequences of emerin dysfunction. Activated STAT3 promotes skeletal muscle atrophy in muscle diseases settings, while functioning as an oncogene in non-muscular cells [33,34,35]. Altered STAT3 activity associated with emerin perturbation may correlate with changes in Pax7 expression and enhanced proliferation during myogenic differentiation. These observations suggest that emerin-dependent modulation of STAT3 signaling may influence muscle stem cell behavior, without constituting a direct causal mechanism for disease onset [68]. Accordingly, EDMD and related nuclear-envelope disorders are increasingly linked to altered regulation of STAT3, Wnt, and Notch signaling pathways that are essential for skeletal muscle cell proliferation and differentiation [67,68,69] (Figure 2).

### 5.3. MAN1, Smad Signaling, and Muscle Atrophy

MAN1 is another member of the LEM domain protein family and an integral components of the nuclear envelope with important implications for muscle physiology [43]. Proper nuclear envelope configuration is essential for muscle integrity, as it supports nuclear mechanics and regulates transcriptional responses to mechanical and biochemical signals, including Smad-mediated pathways [97,120,121].

The Smad signaling pathway, particularly involving Smad2 and Smad3, represents a major downstream effector of TGF-β and myostatin signaling, both of which inhibit muscle growth and promote muscle atrophy [122,123,124]. Myostatin, a key negative regulator of muscle mass, exerts its effects through Smad-dependent induction of muscle-specific ubiquitin ligases like atrogin-1, leading to protein degradation and muscle wasting [124,125]. Consequently, activation of the Smad2/3 pathway is generally associated with negative regulation of muscle mass and atrophic phenotypes [122,123,125,126].

Through its interaction with Smad proteins, MAN1 functions as a modulator of TGF-β/Smad signaling at the nuclear envelope. By spatially restricting Smad activity, MAN1 contributes to the fine-tuning of transcriptional programs that influence muscle mass and tissue homeostasis. Although MAN1 and Smad proteins act through distinct molecular mechanisms, their functional interplay highlights the importance of nuclear envelope-based regulation in muscle maintenance.

### 5.4. Conceptual Integration and Therapeutic Implications

Collectively, these findings support a unifying concept in which LEM-domain proteins act as central organizers that couple nuclear architecture to intranuclear signaling pathways governing muscle development, regeneration, and disease. Rather than acting through single linear pathways, LAP2, emerin, and MAN1 integrate multiple signaling inputs at the nuclear envelope, shaping transcriptional outputs in a tissue- and context-dependent manner.

Understanding how LEM-domain proteins coordinate intranuclear signaling networks provides a conceptual basis for future mechanistic and translational studies in muscle-wasting diseases. Targeting nuclear envelope-associated signaling regulators, including STAT3-, Wnt-, Notch-, and Smad-dependent pathways, may offer new strategies to restore balanced transcriptional control and improve muscle function. Future studies aimed at dissecting LEM-domain protein-mediated signaling across diverse pathophysiological contexts will be essential for translating these insights into effective therapeutic approaches for muscle diseases.

## 6. Conclusions

LEM-domain-containing inner nuclear membrane proteins have emerged as central regulators at the interface between nuclear architecture and signal-dependent transcription. Beyond their classical roles as structural components of the nuclear envelope, proteins such as LAP2, emerin, and MAN1 actively participate in the spatial and temporal control of intranuclear signaling pathways. Collectively, the evidence reviewed here supports a unifying paradigm in which transient sequestration and controlled release of transcriptional regulators at the inner nuclear membrane shape the amplitude, timing, and specificity of gene expression.

While many studies have focused on muscle-related phenotypes, the regulatory principles governed by LEM-domain proteins are not restricted to a single tissue. Instead, the downstream consequences of LEM-domain-mediated signaling diverge across tissues depending on pathway utilization, transcription factor availability, and mechanical context. In skeletal muscle, emerin-dependent coordination of Notch and Wnt signaling contributes to myogenic progression [67,68,119], whereas STAT3 exemplifies context dependence signaling by promoting muscle atrophy in disease settings while functioning as an oncogene in non-muscular cells [33,34,35]. These examples underscore the broader relevance of spatiotemporal gating at the inner nuclear membrane.

Mechanistically, individual LEM-domain proteins regulate distinct but convergent signaling pathways. LAP2 modulates transcriptional activity through dynamic interactions with factors such as GLI1 and β-catenin, emerin spatially restrains signaling mediators including STAT3 and the Notch intracellular domain, and MAN1 fine-tunes TGF-β/Smad signaling at the nuclear periphery. Together, these mechanisms highlight a shared principle whereby nuclear envelope-associated proteins act as regulatory platforms that integrate nuclear structure with signal-dependent transcription, rather than serving as passive scaffolds.

Importantly, dysregulation of LEM-domain-mediated signaling is increasingly recognized as a key contributor to nuclear envelope-associated diseases, particularly muscle-related disorders such as Emery–Dreifuss muscular dystrophy and other laminopathies. The intimate link between nuclear envelope integrity, intranuclear signaling, and muscle homeostasis provides a mechanistic framework for understanding why mechanically active tissues are especially vulnerable to perturbations in nuclear membrane-based regulatory systems, despite the ubiquitous expression of these proteins.

Looking forward, several critical questions remain. These include how signaling-specific interactions are dynamically regulated at the inner nuclear membrane, how LEM-domain proteins coordinate multiple pathways simultaneously, and how mechanical cues intersect with biochemical signaling in the nuclear environment. Addressing these questions will require integrated approaches combining structural biology, advanced imaging, and systems-level analyses.

In conclusion, LEM-domain proteins redefine the nuclear envelope as an active signaling compartment rather than a static boundary. Elucidating the mechanisms by which these proteins integrate nuclear architecture with transcriptional signaling will not only advance our understanding of nuclear biology but also open new avenues for therapeutic strategies targeting nuclear envelope-associated signaling dysfunction in muscle diseases and beyond.

## Data Availability

No new data were created or analyzed in this study. Data sharing is not applicable to this article.

## Abbreviations

The following abbreviations are used in this manuscript:

Hh	Hedgehog
SMO	Smoothened
SUFU	Suppressor of Fused
GLI1	Glioma-associated oncogene 1
LRP	low-density lipoprotein receptor-related protein
GSK-3β	glycogen synthase kinase 3 beta
CKIα	casein kinase I alpha
APC	adenomatous polyposis coli
TCF/LEF	T-cell factor/lymphoid enhancer factor
JAK	Janus kinase
STAT3	signal transducer and activator of transcription 3
ADAM	a disintegrin and metalloprotease
NICD	Notch intracellular domain
Co-A	co-activators
MAML	mastermind-like
CSL	CBF1/Suppressor of Hairless/LAG-1
TGF-β	transforming growth factor beta
Smad, SMA	small body size, *C. elegans*
MAD	mothers against decapentaplegic, *Drosophila*

## References

[1] Liebman C., McColloch A., Rabiei M., Bowling A., Cho M. (2020). Mechanics of the cell: Interaction mechanisms and mechanobiological models. Curr. Top. Membr..

[2] Barton L.J., Soshnev A.A., Geyer P.K. (2015). Networking in the nucleus: A spotlight on LEM-domain proteins. Curr. Opin. Cell Biol..

[3] Wagner N., Krohne G. (2007). LEM-Domain proteins: New insights into lamin-interacting proteins. Int. Rev. Cytol..

[4] Dultz E., Ellenberg J. (2007). Nuclear envelope. Curr. Biol..

[5] Dauer W.T., Worman H.J. (2009). The nuclear envelope as a signaling node in development and disease. Dev. Cell.

[6] Wong X., Melendez-Perez A.J., Reddy K.L. (2022). The Nuclear Lamina. Cold Spring Harb. Perspect. Biol..

[7] Dechat T., Pfleghaar K., Sengupta K., Shimi T., Shumaker D.K., Solimando L., Goldman R.D. (2008). Nuclear lamins: Major factors in the structural organization and function of the nucleus and chromatin. Genes Dev..

[8] Flores L.F., Tader B.R., Tolosa E.J., Sigafoos A.N., Marks D.L., Fernandez-Zapico M.E. (2021). Nuclear Dynamics and Chromatin Structure: Implications for Pancreatic Cancer. Cells.

[9] Berk J.M., Tifft K.E., Wilson K.L. (2013). The nuclear envelope LEM-domain protein emerin. Nucleus.

[10] Sokpor G., Xie Y., Rosenbusch J., Tuoc T. (2017). Chromatin Remodeling BAF (SWI/SNF) Complexes in Neural Development and Disorders. Front. Mol. Neurosci..

[11] Walters A.D., Cohen-Fix O. (2013). Nuclear division: Giving daughters their fair share. Curr. Biol..

[12] Guttinger S., Laurell E., Kutay U. (2009). Orchestrating nuclear envelope disassembly and reassembly during mitosis. Nat. Rev. Mol. Cell Biol..

[13] Laguri C., Gilquin B., Wolff N., Romi-Lebrun R., Courchay K., Callebaut I., Worman H.J., Zinn-Justin S. (2001). Structural characterization of the LEM motif common to three human inner nuclear membrane proteins. Structure.

[14] Bradley C.M., Jones S., Huang Y., Suzuki Y., Kvaratskhelia M., Hickman A.B., Craigie R., Dyda F. (2007). Structural basis for dimerization of LAP2alpha, a component of the nuclear lamina. Structure.

[15] Samson C., Petitalot A., Celli F., Herrada I., Ropars V., Le Du M.H., Nhiri N., Jacquet E., Arteni A.A., Buendia B. (2018). Structural analysis of the ternary complex between lamin A/C, BAF and emerin identifies an interface disrupted in autosomal recessive progeroid diseases. Nucleic Acids Res..

[16] Miyazono K.I., Ohno Y., Wada H., Ito T., Fukatsu Y., Kurisaki A., Asashima M., Tanokura M. (2018). Structural basis for receptor-regulated SMAD recognition by MAN1. Nucleic Acids Res..

[17] Konde E., Bourgeois B., Tellier-Lebegue C., Wu W., Perez J., Caputo S., Attanda W., Gasparini S., Charbonnier J.B., Gilquin B. (2010). Structural analysis of the Smad2-MAN1 interaction that regulates transforming growth factor-beta signaling at the inner nuclear membrane. Biochemistry.

[18] Andres V., Gonzalez J.M. (2009). Role of A-type lamins in signaling, transcription, and chromatin organization. J. Cell Biol..

[19] Dorner D., Gotzmann J., Foisner R. (2007). Nucleoplasmic lamins and their interaction partners, LAP2alpha, Rb, and BAF, in transcriptional regulation. FEBS J..

[20] Tang Y., Zhang X., Ge W., Zhou Y. (2020). Knockdown of LAP2alpha inhibits osteogenic differentiation of human adipose-derived stem cells by activating NF-kappaB. Stem Cell Res. Ther..

[21] Hernandez-Hernandez J.M., Garcia-Gonzalez E.G., Brun C.E., Rudnicki M.A. (2017). The myogenic regulatory factors, determinants of muscle development, cell identity and regeneration. Semin. Cell Dev. Biol..

[22] Martins S.G., Ribeiro V., Melo C., Paulino-Cavaco C., Antonini D., Dayalan Naidu S., Murtinheira F., Fonseca I., Saget B., Pita M. (2024). Laminin-alpha2 chain deficiency in skeletal muscle causes dysregulation of multiple cellular mechanisms. Life Sci. Alliance.

[23] Yanay N., Rabie M., Nevo Y. (2020). Impaired Regeneration in Dystrophic Muscle-New Target for Therapy. Front. Mol. Neurosci..

[24] Mann C.J., Perdiguero E., Kharraz Y., Aguilar S., Pessina P., Serrano A.L., Munoz-Canoves P. (2011). Aberrant repair and fibrosis development in skeletal muscle. Skelet. Muscle.

[25] Maggi L., Carboni N., Bernasconi P. (2016). Skeletal Muscle Laminopathies: A Review of Clinical and Molecular Features. Cells.

[26] Batista N.J., Desai S.G., Perez A.M., Finkelstein A., Radigan R., Singh M., Landman A., Drittel B., Abramov D., Ahsan M. (2023). The Molecular and Cellular Basis of Hutchinson-Gilford Progeria Syndrome and Potential Treatments. Genes.

[27] Lamis A., Siddiqui S.W., Ashok T., Patni N., Fatima M., Aneef A.N. (2022). Hutchinson-Gilford Progeria Syndrome: A Literature Review. Cureus.

[28] Takamizawa K., Kim K.S., Ueda H. (2022). Emery-Dreifuss muscular dystrophy with dilated cardiomyopathy preceding skeletal muscle symptoms. Cardiol. Young.

[29] Ross J.A., Arcos-Villacis N., Battey E., Boogerd C., Orellana C.A., Marhuenda E., Swiatlowska P., Hodzic D., Prin F., Mohun T. (2023). Lem2 is essential for cardiac development by maintaining nuclear integrity. Cardiovasc. Res..

[30] Kovalchuk T., Yakovleva E., Fetisova S., Vershinina T., Lebedeva V., Lyubimtseva T., Lebedev D., Mitrofanova L., Ryzhkov A., Sokolnikova P. (2021). Case Reports: Emery-Dreifuss Muscular Dystrophy Presenting as a Heart Rhythm Disorders in Children. Front. Cardiovasc. Med..

[31] Caravia X.M., Ramirez-Martinez A., Gan P., Wang F., McAnally J.R., Xu L., Bassel-Duby R., Liu N., Olson E.N. (2022). Loss of function of the nuclear envelope protein LEMD2 causes DNA damage-dependent cardiomyopathy. J. Clin. Investig..

[32] Bulmer L., Ljungman C., Hallin J., Dahlberg P., Polte C.L., Hedberg-Oldfors C., Oldfors A., Gummesson A. (2025). EMD missense variant causes X-linked isolated dilated cardiomyopathy with myocardial emerin deficiency. Eur. J. Hum. Genet..

[33] Nunes A.M., Wuebbles R.D., Sarathy A., Fontelonga T.M., Deries M., Burkin D.J., Thorsteinsdottir S. (2017). Impaired fetal muscle development and JAK-STAT activation mark disease onset and progression in a mouse model for merosin-deficient congenital muscular dystrophy. Hum. Mol. Genet..

[34] Bromberg J.F., Wrzeszczynska M.H., Devgan G., Zhao Y., Pestell R.G., Albanese C., Darnell J.E. (1999). Stat3 as an oncogene. Cell.

[35] Wake M.S., Watson C.J. (2015). STAT3 the oncogene—Still eluding therapy?. FEBS J..

[36] Cohen T.V., Kosti O., Stewart C.L. (2007). The nuclear envelope protein MAN1 regulates TGFbeta signaling and vasculogenesis in the embryonic yolk sac. Development.

[37] Osada S., Ohmori S.Y., Taira M. (2003). XMAN1, an inner nuclear membrane protein, antagonizes BMP signaling by interacting with Smad1 in Xenopus embryos. Development.

[38] Moser B., Basilio J., Gotzmann J., Brachner A., Foisner R. (2020). Comparative Interactome Analysis of Emerin, MAN1 and LEM2 Reveals a Unique Role for LEM2 in Nucleotide Excision Repair. Cells.

[39] Gomez-Cavazos J.S., Hetzer M.W. (2012). Outfits for different occasions: Tissue-specific roles of Nuclear Envelope proteins. Curr. Opin. Cell Biol..

[40] Broers J.L., Ramaekers F.C., Bonne G., Yaou R.B., Hutchison C.J. (2006). Nuclear lamins: Laminopathies and their role in premature ageing. Physiol. Rev..

[41] Sidorenko E., Sokolova M., Pennanen A.P., Kyheroinen S., Posern G., Foisner R., Vartiainen M.K. (2022). Lamina-associated polypeptide 2alpha is required for intranuclear MRTF-A activity. Sci. Rep..

[42] Somech R., Shaklai S., Geller O., Amariglio N., Simon A.J., Rechavi G., Gal-Yam E.N. (2005). The nuclear-envelope protein and transcriptional repressor LAP2beta interacts with HDAC3 at the nuclear periphery, and induces histone H4 deacetylation. J. Cell Sci..

[43] Dechat T., Vlcek S., Foisner R. (2000). Review: Lamina-associated polypeptide 2 isoforms and related proteins in cell cycle-dependent nuclear structure dynamics. J. Struct. Biol..

[44] Gesson K., Vidak S., Foisner R. (2014). Lamina-associated polypeptide (LAP)2alpha and nucleoplasmic lamins in adult stem cell regulation and disease. Semin. Cell Dev. Biol..

[45] Gant T.M., Harris C.A., Wilson K.L. (1999). Roles of LAP2 proteins in nuclear assembly and DNA replication: Truncated LAP2beta proteins alter lamina assembly, envelope formation, nuclear size, and DNA replication efficiency in Xenopus laevis extracts. J. Cell Biol..

[46] Shumaker D.K., Lee K.K., Tanhehco Y.C., Craigie R., Wilson K.L. (2001). LAP2 binds to BAF.DNA complexes: Requirement for the LEM domain and modulation by variable regions. EMBO J..

[47] Martins S., Eikvar S., Furukawa K., Collas P. (2003). HA95 and LAP2 beta mediate a novel chromatin-nuclear envelope interaction implicated in initiation of DNA replication. J. Cell Biol..

[48] Martins S.B., Marstad A., Collas P. (2003). In vitro modulation of the interaction between HA95 and LAP2beta by cAMP signaling. Biochemistry.

[49] Zheng R., Ghirlando R., Lee M.S., Mizuuchi K., Krause M., Craigie R. (2000). Barrier-to-autointegration factor (BAF) bridges DNA in a discrete, higher-order nucleoprotein complex. Proc. Natl. Acad. Sci. USA.

[50] Ji S.J., Jaffrey S.R. (2014). Axonal transcription factors: Novel regulators of growth cone-to-nucleus signaling. Dev. Neurobiol..

[51] Mirza A.N., McKellar S.A., Urman N.M., Brown A.S., Hollmig T., Aasi S.Z., Oro A.E. (2019). LAP2 Proteins Chaperone GLI1 Movement between the Lamina and Chromatin to Regulate Transcription. Cell.

[52] Mirza A.N., Gonzalez F., Ha S.K., Oro A.E. (2021). The Sky’s the LEMit: New insights into nuclear structure regulation of transcription factor activity. Curr. Opin. Cell Biol..

[53] Milazzo G., Mercatelli D., Di Muzio G., Triboli L., De Rosa P., Perini G., Giorgi F.M. (2020). Histone Deacetylases (HDACs): Evolution, Specificity, Role in Transcriptional Complexes, and Pharmacological Actionability. Genes.

[54] Doheny D., Manore S.G., Wong G.L., Lo H.W. (2020). Hedgehog Signaling and Truncated GLI1 in Cancer. Cells.

[55] Avery J.T., Zhang R., Boohaker R.J. (2021). GLI1: A Therapeutic Target for Cancer. Front. Oncol..

[56] Wang W., Yan T., Guo W., Niu J., Zhao Z., Sun K., Zhang H., Yu Y., Ren T. (2021). Constitutive GLI1 expression in chondrosarcoma is regulated by major vault protein via mTOR/S6K1 signaling cascade. Cell Death Differ..

[57] Peng J., Han L., Liu B., Song J., Wang Y., Wang K., Guo Q., Liu X., Li Y., Zhang J. (2023). Gli1 marks a sentinel muscle stem cell population for muscle regeneration. Nat. Commun..

[58] Norris A.M., Appu A.B., Johnson C.D., Zhou L.Y., McKellar D.W., Renault M.A., Hammers D., Cosgrove B.D., Kopinke D. (2023). Hedgehog signaling via its ligand DHH acts as cell fate determinant during skeletal muscle regeneration. Nat. Commun..

[59] Markiewicz E., Dechat T., Foisner R., Quinlan R.A., Hutchison C.J. (2002). Lamin A/C binding protein LAP2alpha is required for nuclear anchorage of retinoblastoma protein. Mol. Biol. Cell.

[60] Dorner D., Vlcek S., Foeger N., Gajewski A., Makolm C., Gotzmann J., Hutchison C.J., Foisner R. (2006). Lamina-associated polypeptide 2alpha regulates cell cycle progression and differentiation via the retinoblastoma-E2F pathway. J. Cell Biol..

[61] Pekovic V., Harborth J., Broers J.L., Ramaekers F.C., van Engelen B., Lammens M., von Zglinicki T., Foisner R., Hutchison C., Markiewicz E. (2007). Nucleoplasmic LAP2alpha-lamin A complexes are required to maintain a proliferative state in human fibroblasts. J. Cell Biol..

[62] Vlcek S., Korbei B., Foisner R. (2002). Distinct functions of the unique C terminus of LAP2alpha in cell proliferation and nuclear assembly. J. Biol. Chem..

[63] Ferraioli S., Sarigol F., Prakash C., Filipczak D., Foisner R., Naetar N. (2024). LAP2alpha facilitates myogenic gene expression by preventing nucleoplasmic lamin A/C from spreading to active chromatin regions. Nucleic Acids Res..

[64] Shin J.Y., Worman H.J. (2022). Molecular Pathology of Laminopathies. Annu. Rev. Pathol..

[65] Naetar N., Korbei B., Kozlov S., Kerenyi M.A., Dorner D., Kral R., Gotic I., Fuchs P., Cohen T.V., Bittner R. (2008). Loss of nucleoplasmic LAP2alpha-lamin A complexes causes erythroid and epidermal progenitor hyperproliferation. Nat. Cell Biol..

[66] Gotic I., Schmidt W.M., Biadasiewicz K., Leschnik M., Spilka R., Braun J., Stewart C.L., Foisner R. (2010). Loss of LAP2 alpha delays satellite cell differentiation and affects postnatal fiber-type determination. Stem Cells.

[67] Lee B., Lee T.H., Shim J. (2017). Emerin suppresses Notch signaling by restricting the Notch intracellular domain to the nuclear membrane. Biochim. Biophys. Acta Mol. Cell Res..

[68] Lee B., Lee S., Lee Y., Park Y., Shim J. (2021). Emerin Represses STAT3 Signaling through Nuclear Membrane-Based Spatial Control. Int. J. Mol. Sci..

[69] Markiewicz E., Tilgner K., Barker N., van de Wetering M., Clevers H., Dorobek M., Hausmanowa-Petrusewicz I., Ramaekers F.C., Broers J.L., Blankesteijn W.M. (2006). The inner nuclear membrane protein emerin regulates beta-catenin activity by restricting its accumulation in the nucleus. EMBO J..

[70] Dedeic Z., Cetera M., Cohen T.V., Holaska J.M. (2011). Emerin inhibits Lmo7 binding to the Pax3 and MyoD promoters and expression of myoblast proliferation genes. J. Cell Sci..

[71] Holaska J.M., Lee K.K., Kowalski A.K., Wilson K.L. (2003). Transcriptional repressor germ cell-less (GCL) and barrier to autointegration factor (BAF) compete for binding to emerin in vitro. J. Biol. Chem..

[72] Holaska J.M., Wilson K.L. (2006). Multiple roles for emerin: Implications for Emery-Dreifuss muscular dystrophy. Anat. Rec. A Discov. Mol. Cell Evol. Biol..

[73] Wilkinson F.L., Holaska J.M., Zhang Z., Sharma A., Manilal S., Holt I., Stamm S., Wilson K.L., Morris G.E. (2003). Emerin interacts in vitro with the splicing-associated factor, YT521-B. Eur. J. Biochem..

[74] Koch A.J., Holaska J.M. (2014). Emerin in health and disease. Semin. Cell Dev. Biol..

[75] Cho S., Irianto J., Discher D.E. (2017). Mechanosensing by the nucleus: From pathways to scaling relationships. J. Cell Biol..

[76] Maurer M., Lammerding J. (2019). The Driving Force: Nuclear Mechanotransduction in Cellular Function, Fate, and Disease. Annu. Rev. Biomed. Eng..

[77] Miroshnikova Y.A., Wickstrom S.A. (2022). Mechanical Forces in Nuclear Organization. Cold Spring Harb. Perspect. Biol..

[78] Nastaly P., Purushothaman D., Marchesi S., Poli A., Lendenmann T., Kidiyoor G.R., Beznoussenko G.V., Lavore S., Romano O.M., Poulikakos D. (2020). Role of the nuclear membrane protein Emerin in front-rear polarity of the nucleus. Nat. Commun..

[79] Isermann P., Lammerding J. (2013). Nuclear mechanics and mechanotransduction in health and disease. Curr. Biol..

[80] Hildebrand E.M., Dekker J. (2020). Mechanisms and Functions of Chromosome Compartmentalization. Trends Biochem. Sci..

[81] Kim Y., Zheng X., Zheng Y. (2019). Role of lamins in 3D genome organization and global gene expression. Nucleus.

[82] Katta S.S., Smoyer C.J., Jaspersen S.L. (2014). Destination: Inner nuclear membrane. Trends Cell Biol..

[83] Pawar S., Kutay U. (2021). The Diverse Cellular Functions of Inner Nuclear Membrane Proteins. Cold Spring Harb. Perspect. Biol..

[84] Yu H., Jove R. (2004). The STATs of cancer--new molecular targets come of age. Nat. Rev. Cancer.

[85] Kanda N., Seno H., Konda Y., Marusawa H., Kanai M., Nakajima T., Kawashima T., Nanakin A., Sawabu T., Uenoyama Y. (2004). STAT3 is constitutively activated and supports cell survival in association with survivin expression in gastric cancer cells. Oncogene.

[86] Debnath B., Xu S., Neamati N. (2012). Small molecule inhibitors of signal transducer and activator of transcription 3 (Stat3) protein. J. Med. Chem..

[87] Lopez-Soler R.I., Moir R.D., Spann T.P., Stick R., Goldman R.D. (2001). A role for nuclear lamins in nuclear envelope assembly. J. Cell Biol..

[88] Odell J., Lammerding J. (2023). Lamins as structural nuclear elements through evolution. Curr. Opin. Cell Biol..

[89] Puente X.S., Quesada V., Osorio F.G., Cabanillas R., Cadinanos J., Fraile J.M., Ordonez G.R., Puente D.A., Gutierrez-Fernandez A., Fanjul-Fernandez M. (2011). Exome sequencing and functional analysis identifies BANF1 mutation as the cause of a hereditary progeroid syndrome. Am. J. Hum. Genet..

[90] Jamin A., Wiebe M.S. (2015). Barrier to Autointegration Factor (BANF1): Interwoven roles in nuclear structure, genome integrity, innate immunity, stress responses and progeria. Curr. Opin. Cell Biol..

[91] Burgess J.T., Cheong C.M., Suraweera A., Sobanski T., Beard S., Dave K., Rose M., Boucher D., Croft L.V., Adams M.N. (2021). Barrier-to-autointegration-factor (Banf1) modulates DNA double-strand break repair pathway choice via regulation of DNA-dependent kinase (DNA-PK) activity. Nucleic Acids Res..

[92] Pradhan R., Ranade D., Sengupta K. (2018). Emerin modulates spatial organization of chromosome territories in cells on softer matrices. Nucleic Acids Res..

[93] Iyer A., Holaska J.M. (2020). EDMD-Causing Emerin Mutant Myogenic Progenitors Exhibit Impaired Differentiation Using Similar Mechanisms. Cells.

[94] Lee G.E., Byun J., Lee C.J., Cho Y.Y. (2023). Molecular Mechanisms for the Regulation of Nuclear Membrane Integrity. Int. J. Mol. Sci..

[95] Lin F., Blake D.L., Callebaut I., Skerjanc I.S., Holmer L., McBurney M.W., Paulin-Levasseur M., Worman H.J. (2000). MAN1, an inner nuclear membrane protein that shares the LEM domain with lamina-associated polypeptide 2 and emerin. J. Biol. Chem..

[96] Gruenbaum Y., Margalit A., Goldman R.D., Shumaker D.K., Wilson K.L. (2005). The nuclear lamina comes of age. Nat. Rev. Mol. Cell Biol..

[97] Bengtsson L. (2007). What MAN1 does to the Smads. TGFbeta/BMP signaling and the nuclear envelope. FEBS J..

[98] Furler R.L., Nixon D.F., Brantner C.A., Popratiloff A., Uittenbogaart C.H. (2018). TGF-beta Sustains Tumor Progression through Biochemical and Mechanical Signal Transduction. Cancers.

[99] Pan D., Estevez-Salmeron L.D., Stroschein S.L., Zhu X., He J., Zhou S., Luo K. (2005). The integral inner nuclear membrane protein MAN1 physically interacts with the R-Smad proteins to repress signaling by the transforming growth factor-beta superfamily of cytokines. J. Biol. Chem..

[100] Kubiczkova L., Sedlarikova L., Hajek R., Sevcikova S. (2012). TGF-beta—An excellent servant but a bad master. J. Transl. Med..

[101] Deng Z., Fan T., Xiao C., Tian H., Zheng Y., Li C., He J. (2024). TGF-beta signaling in health, disease, and therapeutics. Signal Transduct. Target. Ther..

[102] Chen P.Y., Qin L., Simons M. (2023). TGFbeta signaling pathways in human health and disease. Front. Mol. Biosci..

[103] Bourgeois B., Gilquin B., Tellier-Lebegue C., Ostlund C., Wu W., Perez J., El Hage P., Lallemand F., Worman H.J., Zinn-Justin S. (2013). Inhibition of TGF-beta signaling at the nuclear envelope: Characterization of interactions between MAN1, Smad2 and Smad3, and PPM1A. Sci. Signal.

[104] Melchionna R., Trono P., Tocci A., Nistico P. (2021). Actin Cytoskeleton and Regulation of TGFbeta Signaling: Exploring Their Links. Biomolecules.

[105] Liu J., Lee K.K., Segura-Totten M., Neufeld E., Wilson K.L., Gruenbaum Y. (2003). MAN1 and emerin have overlapping function(s) essential for chromosome segregation and cell division in Caenorhabditis elegans. Proc. Natl. Acad. Sci. USA.

[106] Rose M., Burgess J.T., O’Byrne K., Richard D.J., Bolderson E. (2022). The role of inner nuclear membrane proteins in tumourigenesis and as potential targets for cancer therapy. Cancer Metastasis Rev..

[107] Janin A., Bauer D., Ratti F., Millat G., Mejat A. (2017). Nuclear envelopathies: A complex LINC between nuclear envelope and pathology. Orphanet J. Rare Dis..

[108] Gotic I., Foisner R. (2010). Multiple novel functions of lamina associated polypeptide 2alpha in striated muscle. Nucleus.

[109] Worman H.J., Bonne G. (2007). “Laminopathies”: A wide spectrum of human diseases. Exp. Cell Res..

[110] Kalukula Y., Stephens A.D., Lammerding J., Gabriele S. (2022). Mechanics and functional consequences of nuclear deformations. Nat. Rev. Mol. Cell Biol..

[111] Zhang B., Powers J.D., McCulloch A.D., Chi N.C. (2023). Nuclear mechanosignaling in striated muscle diseases. Front. Physiol..

[112] Ellis J.A., Craxton M., Yates J.R., Kendrick-Jones J. (1998). Aberrant intracellular targeting and cell cycle-dependent phosphorylation of emerin contribute to the Emery-Dreifuss muscular dystrophy phenotype. J. Cell Sci..

[113] Ostlund C., Ellenberg J., Hallberg E., Lippincott-Schwartz J., Worman H.J. (1999). Intracellular trafficking of emerin, the Emery-Dreifuss muscular dystrophy protein. J. Cell Sci..

[114] Ostlund C., Sullivan T., Stewart C.L., Worman H.J. (2006). Dependence of diffusional mobility of integral inner nuclear membrane proteins on A-type lamins. Biochemistry.

[115] Eriksson M., Brown W.T., Gordon L.B., Glynn M.W., Singer J., Scott L., Erdos M.R., Robbins C.M., Moses T.Y., Berglund P. (2003). Recurrent de novo point mutations in lamin A cause Hutchinson-Gilford progeria syndrome. Nature.

[116] Goldman R.D., Shumaker D.K., Erdos M.R., Eriksson M., Goldman A.E., Gordon L.B., Gruenbaum Y., Khuon S., Mendez M., Varga R. (2004). Accumulation of mutant lamin A causes progressive changes in nuclear architecture in Hutchinson-Gilford progeria syndrome. Proc. Natl. Acad. Sci. USA.

[117] Astejada M.N., Goto K., Nagano A., Ura S., Noguchi S., Nonaka I., Nishino I., Hayashi Y.K. (2007). Emerinopathy and laminopathy clinical, pathological and molecular features of muscular dystrophy with nuclear envelopathy in Japan. Acta Myol..

[118] Brull A., Morales Rodriguez B., Bonne G., Muchir A., Bertrand A.T. (2018). The Pathogenesis and Therapies of Striated Muscle Laminopathies. Front. Physiol..

[119] Brack A.S., Conboy I.M., Conboy M.J., Shen J., Rando T.A. (2008). A temporal switch from notch to Wnt signaling in muscle stem cells is necessary for normal adult myogenesis. Cell Stem Cell.

[120] Lin F., Morrison J.M., Wu W., Worman H.J. (2005). MAN1, an integral protein of the inner nuclear membrane, binds Smad2 and Smad3 and antagonizes transforming growth factor-beta signaling. Hum. Mol. Genet..

[121] Worman H.J. (2006). Inner nuclear membrane and regulation of Smad-mediated signaling. Biochim. Biophys. Acta.

[122] Sartori R., Milan G., Patron M., Mammucari C., Blaauw B., Abraham R., Sandri M. (2009). Smad2 and 3 transcription factors control muscle mass in adulthood. Am. J. Physiol. Cell Physiol..

[123] Tando T., Hirayama A., Furukawa M., Sato Y., Kobayashi T., Funayama A., Kanaji A., Hao W., Watanabe R., Morita M. (2016). Smad2/3 Proteins Are Required for Immobilization-induced Skeletal Muscle Atrophy. J. Biol. Chem..

[124] Sartori R., Romanello V., Sandri M. (2021). Mechanisms of muscle atrophy and hypertrophy: Implications in health and disease. Nat. Commun..

[125] Salemi S., Schori L.J., Gerwinn T., Horst M., Eberli D. (2023). Myostatin Overexpression and Smad Pathway in Detrusor Derived from Pediatric Patients with End-Stage Lower Urinary Tract Dysfunction. Int. J. Mol. Sci..

[126] Umezu T., Nakamura S., Sato Y., Kobayashi T., Ito E., Abe T., Kaneko M., Nomura M., Yoshimura A., Oya A. (2021). Smad2 and Smad3 expressed in skeletal muscle promote immobilization-induced bone atrophy in mice. Biochem. Biophys. Res. Commun..

